# The SPIN framework to control and prevent the Marburg virus disease outbreak in Equatorial Guinea

**DOI:** 10.11604/pamj.2023.44.110.39368

**Published:** 2023-02-28

**Authors:** Frankline Sevidzem Wirsiy, Claude Ngwayu Nkfusai, Luchuo Engelbert Bain

**Affiliations:** 1 Africa Centres for Disease Control and Prevention (Africa CDC), Addis Ababa, Ethiopia,; 2 Amref Health Africa, Nairobi, Kenya,; 3 Department of Epidemiology, College of Public Health, University of Nebraska Medical Center, Omaha, NE, USA,; 4 Department of Rehabilitation Science and Technology, School of Health and Rehabilitation Sciences, University of Pittsburgh, Pennsylvania, USA,; 5 Department of Public Health, School of Nursing and Public Health, University of Kwa-Zulu Natal, Durban, South Africa,; 6 Malaria Consortium, Buea, Cameroon,; 7 Department of Psychology, Faculty of Humanities, University of Johannesburg, Johannesburg, Auckland Park, South Africa

**Keywords:** Marburg virus, outbreak, Equatorial Guinea, SPIN framework, health security

## Abstract

A full grasp of the epidemiological factors promoting transmission is necessary for responding to highly infectious diseases, which involves their control and prevention. With the recent outbreak of Marburg Virus Disease (MVD) in Equatorial Guinea, we saw the need to re-shed some technical light based on our field experiences and published literature. We reviewed 15 previous MVD outbreaks globally. Coupled with core One-Health approaches, we highlighted the SPIN (socio-environmental context, possible transmission routes, informing and guiding public health action, needs in terms of control measures) framework as a guiding tool for response teams to appropriately approach this highly contagious infectious disease outbreak for collective and stronger global health security. The Central African Regional Collaborating Centre (RCC) of the Africa Centres for Disease Control and Prevention (Africa CDC) has a big lead role to play, most especially in coordinating the community engagement and risk communication packages of the response, which is highly needed at this point. We reiterate that this framework remains relevant, if not timely, in rethinking pandemic preparedness and response in resource-limited settings.

## Commentary

A full grasp of the epidemiological factors promoting transmission is necessary for responding to highly infectious diseases, which involves their control and prevention [1]. The case fatality ratio (CFR; 24-88%) of the epidemic-prone disease Marburg Virus Disease (MVD) is high and due to the similarity of the clinical signs, it can be challenging to identify the early stages of MVD from many other tropical hemorrhagic fever disorders [2]. On February 13^th^, 2023, the first-ever MVD outbreak in Equatorial Guinea was confirmed [3]. Initial tests conducted after at least nine people died in the western Kie Ntem Province of the nation tested positive for viral hemorrhagic fever caused by the Marburg virus (MARV) [3]. It has been determined that the African fruit bat (*Rousettus aegyptiacus*) is the reservoir host for the Marburg virus; a genetically unique zoonotic disease [4]. Humans can become infected with the Marburg virus, and following the discovery of an unidentified hemorrhagic fever last week in its Kie-Ntem district in Equatorial Guinea, more than 200 individuals were isolated and limited travel [5]. Neighboring Cameroon has imposed travel restrictions along its border because on Tuesday 14^th^ February, two suspected cases of MVD were reported in Olamze [6], a Cameroonian commune on the border with Equatorial Guinea; all part of the South Eastern Equatorial rain forest [7,8]. It´s worth noting that, villages of the Kie-Ntem district in Equatorial Guinea are neighboring three health districts of the South region of Cameroon namely, Ambam, Kye-Ossi, and Olamze. This is a stark reminder of the PADRE merits of implementing context-specific National action plans for Health Security (NAPHS) in the Central African region [9].

**Figure 1:**
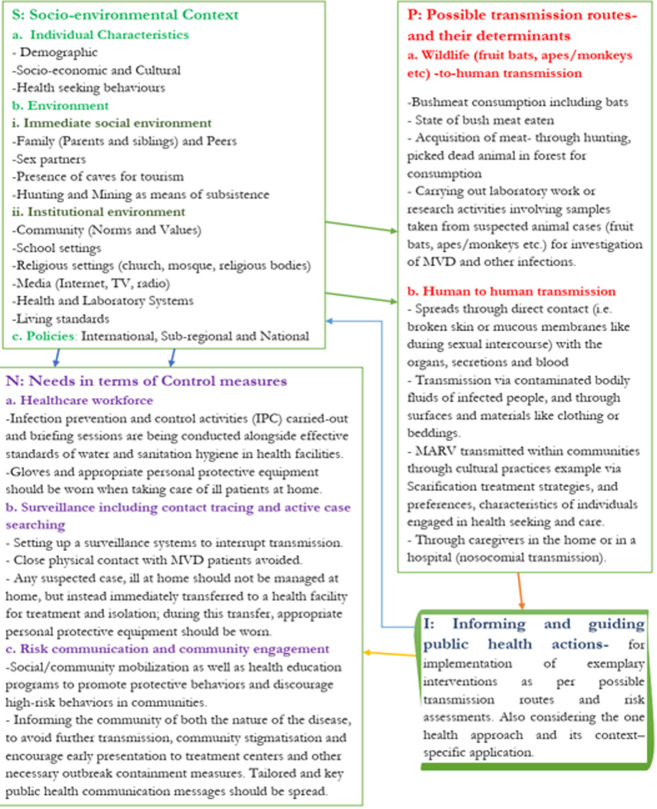
a SPIN framework of transmission and control measures of an MVD outbreak

The Congo Basin, where Equatorial Guinea is located, contains one of the biggest and most biologically diversified rainforests in the world. It has a sizable population of nonhuman primates in the forest, as well as other animals that are prospective and real human pathogen reservoirs. Multiple arthropod-borne viruses, including Chikungunya, Zika, Usutu, and Crimean-Congo hemorrhagic fever virus, Mpox, Ebola, and a recent rhabdovirus named Bas-Congo virus, as well as several significant emerging human viruses, including HIV, are thought to have originated from this region [10]. As of now, there have been a total of 16 MVD outbreaks as seen in Table 1. We propose the SPIN (Socio-environmental context, Possible transmission routes, Informing and guiding public health action, needs in terms of control measures) framework [11] (Figure 1) in consideration of the history of 16 MVD outbreaks that have been recorded which including the context involving the individual characteristics of target communities, the environment in which they find themselves and the policies in place that directly/indirectly affects their needs (control and prevention measures). The SPIN framework is composed of four key elements i.e. the Socio-environmental context (made up of individual characteristics, environment, policies); Possible transmission routes, and their determinants (made up of Wildlife (fruit bats, apes/monkeys, etc.) -to-human transmission and Human to human transmission); Informing and guiding public health actions Needs for implementation of exemplary interventions and control measures (Figure 1).

**Table 1:** sixteen Marburg virus disease outbreaks (1967 to 2023)

No	Year and Country	Suspected and/or Apparent origin	The apparent situation of MVD infection (sociodemographic characteristics, circumstances warranting suspicion, diagnosis, and source of MVD infection)
1.	2023.,Equatorial Guinea	Kie-Ntem province, Equatorial Guinea	Equatorial Guinea on Feb. 13^th^ 2023 confirmed its first-ever outbreak of Marburg virus disease. Preliminary tests carried out following the deaths of at least nine people in the country’s western Kie Ntem Province turned out positive for the viral haemorrhagic fever. The deaths were preliminarily linked to a funeral ceremony in the Kie-Ntem province's Nsok Nsomo district, Equatorial Guinea
2.	2022, Ghana	Ashanti Region, Ghana	A fatal suspect case of Marburg virus disease (MVD) was identified in the Ashanti region of Ghana on July 7, 2022 at Ghana’s national laboratory by polymerase chain reaction (PCR) and confirmed at the Institute Pasteur in Dakar, Senegal. Shortly after, two additional family members were also confirmed to have MVD. No additional cases outside the family cluster were identified.
3.	2021, Guinea	Guéckédou, Guinea	A male, had onset of symptoms in a small health facility near his village of residence with symptoms of fever, headache, fatigue, abdominal pain, and gingival hemorrhage. The patient received supportive care and eventually died in the community. The team collected a post-mortem oral swab sample, for conducting real-time PCR which confirmed the sample was positive for MVD
4.	2017, Uganda	Kween, Uganda	A blood sample from a patient in Kween District in Eastern Uganda tested positive for MARV. Within 24 hours of confirmation, a rapid outbreak response was begun. The index case was a herdsman who hunted games around caves harboring enormous populations of the Egyptian fruit bats
5.	2014, Uganda	Kampala, Uganda	Overall, one case was confirmed (fatal) and 197 contacts were followed for 3 weeks but all tested negative at the Uganda Virus Research Institute (UVRI).
6.	2012, Uganda	Kabale, Uganda	Testing at CDC/UVRI identified a Marburg virus disease outbreak in the districts of Kabale, Ibanda, Mbarara, and Kampala over 3 weeks
7.	2008, Netherlands ex Uganda	Cave in Maramagambo forest in Uganda	A Dutch woman with a recent history of travel to Uganda was admitted to a hospital in the Netherlands. Three days before hospitalization, the first symptoms (fever, chills) occurred, followed by rapid clinical deterioration after which the woman died of MVD
8.	2008, USA ex Uganda	Cave in Maramagambo forest in Uganda	A United States traveler that returned from Uganda was retrospectively diagnosed with MVD.
9.	2007, Uganda	Lead and gold mine in Kamwenge District, Uganda	Four young males working in a mine in Uganda were tested positive for MVD
10.	2004-2005, Angola	Uige Province, Angola	This MVD's largest outbreak is believed to have begun in Uige Province of Angola in October 2004. So far, in the history of MVD outbreaks, this outbreak recorded the highest number of infected cases (252) of which 227 (90%) died. The majority of cases detected in other provinces in Angola have been linked directly to the outbreak in Uige. There was a lag in outbreak identification and poor epidemiological linkage of the cases, resulting in uncertainties as to identifying the origin of the infection
11.	1998-2000, Democratic Republic of Congo (DRC)	Durba, DRC	The MVD cases occurred in young male workers at a gold mine in Durba, in the north-eastern part of the Democratic Republic of Congo
12.	1990, Russia	Russia	The MVD case was a result of laboratory contamination. In this laboratory, there was a non-compliance with safety conditions and/or manipulation error when handling the virus resulting in causing human contamination in Russia in 1990. It is worthy to note that, an infected person remains contagious after his death, thus touching such required appropriate personal protective equipment.
13.	1987, Kenya	Kenya	An MVD infected Danish boy who later died had visited the Kitum Cave in Mount Elgon National Park in Kenya. No further cases were detected.
14.	1980, Kenya	Kenya	The case was that of an MVD male patient that died and had a history of recent travel to Kenya. While in Kenya, he visited the Kitum Cave in Mount Elgon National Park. A doctor who attempted resuscitation developed symptoms 9 days later but recovered.
15.	1975, Johannesburg, South Africa	Zimbabwe	The primary case was in a young Australian man who eventually died with a recent travel history to Zimbabwe for tourism with his spouse. The circumstances regarding their infection suggest that there was likely a direct contact with bats discharge as they slept in rooms containing insectivorous bats which led to the infection. The infection spread from the man to his traveling companion and a nurse at the hospital (nosocomial infection), even though they recovered.
16.	1967, Germany and Yugoslavia	Uganda	Simultaneous outbreaks occurred in laboratory workers handling African green monkeys imported from Uganda. In addition to the 31 reported cases, an additional primary case was retrospectively serologically diagnosed and other cases were reported due to nosocomial transmission. Also, a woman got infected with MVD, 3 months earlier via sexual contact with her husband.

### Risk communication and community engagement

Risk communication and community engagement (RCCE) has been established to be an essential component of emergency preparedness and response [12]. During an outbreak of such caliber, for the acceptance and adoption of life-saving therapies, health information must be reliable, trustworthy, pertinent, timely, accessible, and actionable [13]. The successful engagement of affected people through RCCE strategies and initiatives has proven essential for emergency preparedness and response plans [13]. Ending the MVD outbreak situation requires enhancing confidence through strategic communication and jointly devising solutions that best meet local requirements. To preserve operational readiness for unforeseen events as per the SPIN framework of the control and prevention of this MVD emergency as well as future emergencies, African Member States especially the Central African Regional Collaborating Centre (RCC) of the Africa CDC [14] where Equatorial Guinea is located are advised to put in place and maintain RCCE teams ready to be deployed at the shortest possible time including those at the current emergency levels. To ensure the synchronization of risk communications and community engagement initiatives, cross-border collaboration and coordination across neighboring nations will also need to be improved.

For marginal, vulnerable, and hard-to-reach communities, risk communication is crucial because ignorance kills. The World Health Organization (WHO) has coined the term “infodemic” to describe the overabundance of information often misleading that exists today. Additionally, in situations where risk communication is unclear, rumors might proliferate. It has been proven that low-income and marginalized individuals are frequently left out of top-down risk communication and a study that focused on African nations revealed that the general public does not trust the communication process [15]. Therefore, bottom-top strategies for risk communication and community involvement should also be carefully explored to better control and prevent the MVD outbreak. Given its significance, RCCE ought to be included as a key component of the global health security agenda. To make sure that they are more thorough, the RCCE components of the Joint External Evaluation tools should be reviewed. Additionally, all updated International Health Regulations (IHR) (2005) processes, such as joint external evaluations, joint monitoring missions, joint assessment missions, and after-action reviews, should purposefully include RCCE essential partners.

### Control and prevention of Marburg virus disease

The establishment of event-based surveillance, a good laboratory-systems service, and safe and respectable burials are only a few examples of actions that should be included in a model of an MVD outbreak management strategy. Moreover, setting up a surveillance system to stop transmission, and social and community mobilization including community engagement, as well as health education campaigns to encourage protective behaviors and discourage high-risk behaviors in communities, are crucial methods for controlling an MVD outbreak [4]. Since research on the transmission of the Marburg virus from wildlife to humans is still underway, prevention strategies against infection are not well established [16]. But one method to guard against infection is to stay away from sick non-human primates and fruit bats. Similar to those employed for other hemorrhagic fevers, secondary, or person-to-person, transmission prevention measures are available. Direct physical contact with a patient should be avoided if they have Marburg virus disease (MVD), whether they are suspected of having it or have been diagnosed with it. These safety measures include putting on protective clothing, gloves, and masks; isolating the diseased person; and sterilizing or properly discarding needles, tools, and patient excretions.

## Conclusion

Africa is at the crossroad in safeguarding its health security because more than 1.3 billion people live on the continent of Africa, which continues to have the highest annual incidence of public health emergencies. Over 65% of the more than 1.3 billion people who call Africa home are young people. Young healthcare workers and advocates are behind this figure, and they have a tremendous deal of potential to innovate and improve health security in Africa. They are essential to advancing public health in African nations and interacting with communities; as a result, they serve as partners, resources, and a requirement for the continent's long-term health security. Africa´s health security has been made worse by the harm that ongoing outbreaks and epidemics are causing to Africa's health and socio-economic sectors. Another issue is the difficulty that African Union Member States have in obtaining crucial medical countermeasures during outbreaks due to trade and intellectual property-related restrictions. This notwithstanding there is a revolution dubbed the New Public Health Order established by the Africa Centres for Disease Control (Africa CDC) which should be our inspiration for collective and stronger global health security. Equally, a flexible strategy for controlling and preventing infectious diseases like MVD is the implementation of the One Health approach. Using a One-Health-based strategy to manage an infectious disease has had a positive impact on containing recent outbreaks and more focused efforts should be made to promote knowledge of Social and Structural Influences of Health (SSIH) to assess infectious disease challenges from a One-Health perspective. The true promise of One-Health to reduce infectious disease outbreaks can only be realized through the comprehensive involvement of all relevant field players. This introduces the idea of collaboration/partnership within and or between African governments and institutions, such as the African Union (AU), Africa CDC, WHO African region, Amref Health Africa, etc. to achieve shared public health objectives. To end the acute phase of the COVID-19 pandemic and stop this MVD outbreak in Equatorial Guinea, we must put aside our differences, come together as one because “many heads are better than one,” and work collaboratively. By doing so, we can continue to advance our goals of advancing universal health coverage, strengthening collective and national health security, advancing health equity, strengthening health systems and institutions as well as accelerating efforts to achieve the United Nations 2030 Agenda for Sustainable Development.
